# Royal British Nurses' Association Meeting

**Published:** 1918-11-23

**Authors:** 


### THE REGISTRATION OF TRAINED NURSES.

### Royal British Nurses' Association Meeting.

This was held at 11 Chandos Street, Cavendish Square, W., on the evening of November 7, 1918. Mr. Herbert J. Paterson, F.R.C.S., was in the chair, representatives of the College and of the Central Committee were present, and a long discussion resulted; but the irreconcilables, after repeating many old gibes disposed of times out of number, refused to agree with the College, and nothing of value resulted from the meeting.

Lieut.-Colonel Goodall, M.D., Hon. Secretary to the Committee, who was followed by Mrs. Bedford Fenwick, had nothing new to urge. Their addresses were wordy and ancient sermons preached to boredom. When discussion was invited the meeting began to wake up a little.

### Is Registration Getting Nearer?

Professor Glaister said he had been associated with those who drafted the Central Committee's Bill many years ago, along with Colonel Goodall. He could not say at the moment whether or not he was a member of the Royal British Nurses' Association—he was some years ago. He had also been associated with the College of Nursing, so he confessed to being one who was greatly interested in the subject they were discussing. Along with others he had spent a good deal of time and energy in Scotland in promoting the State registration of nurses, and what he wanted to say was that while they were quarrelling among themselves about minor details, they had not got a step forrarder with registration. They had been spending years in the work, and they had talked a lot about getting nurses registered, but were they a bit nearer? (Cries of "Yes.") Well, he had not discovered that they had got a scrap nearer to their object so far. He was out for the State registration of nurses, and to get it as quickly as he possibly could. He believed that everyone present wanted to be quite fair about the two Bills that were before them, and he would like to say one or two words on the subject because he had the materials at his disposal. Nobody, he was sure, would be more willing to admit than the first two speakers that even the Bill of the Central Committee was like Topsy—it had "growed." He had before him the last edition of the Bill of the Central Committee, and it was plain that it had undergone evolution as every Bill ought to do. In the same way the juvenile Bill of the College of Nursing had had to undergo evolution also, because he supposed both bodies were striving honestly to get the very best Bill that could be got for the nurses. If that be so, there must be some substantial reason for the criticisms which had been passed.

### Notification of Nurses' Deaths.

Something had been said in criticism of that provision in the College Bill for a notification of the death of nurses. They would perhaps be surprised to know that in that they were only following in the lead of the medical profession. When a doctor died it was the duty of the Registrar to notify the death to the Registrar of the General Medical Council, and if there was one thing to commend that clause more than another it was the fact that it had been adopted by the Central Committee in their Bill. They wanted to be perfectly sure that the register was a live register. He was well aware that there were points of dispute in the College Bill—in his opinion a Bill would not be worth having unless it had points of dispute in it. To deal with what had been said about the supplementary registers—he would be the last person to admit anyone on to the register by a back or a side door, and those who had worked with him were, he believed, aware of that fact; but in the College Bill they did not propose to do more than take powers to institute supplemental registers, if the General Council thought proper. There had been a good deal of confusion about the original machinery provided for in the Bill.

### The Nurses and the Council.

The College Bill recognised that there must be a provisional Council to start things, and trying to be absolutely fair it gave the Central Committee the same number of representatives as it had itself, and, in addition, there were some official representatives. They had equal facilities to do the best thing they could for the nurses, and after the provisional Council had been formed they were asked under the College Bill to do one definite thing—to carry out a sub-clause in Clause (a) of Section 5. There was no time limit fixed to this—if the Central Committee wanted a period of a couple of years there was no reason why they should not have it. The new constituency of nurses which would have been accumulating during those two years, plus those who had registered under the College of Nursing, would be able to appoint the General Nursing Council as a united body. By the Bill of the College it was provided first of all that the General Nursing Council should consist of not less than two-thirds of those who should be elected by the nurses who were on the General Register under the Act, and the clause dealing with that point went on to say—" and of the persons so elected four-sixths shall represent England and Wales, one-sixth Scotland, and one-sixth Ireland"; and, secondly, "of the persons elected by the nurses on the General Register to represent England and Wales, Scotland and Ireland respectively, five-sixths shall be nurses on the General Register." He asked them, could there be anything fairer than that?

The Chairman at this point reminded Professor Glaister that he had had his allotted three minutes, and unless the meeting wished him to continue he must ask him to cease speaking.

Professor Glaister said he had come all the way from Scotland to attend the meeting, and if he had known that he would only have had three minutes he would not have gone into detail. He could only bow to the Chairman's ruling, but he would like to say before he sat down that he hoped nurses would be under no delusion in the matter. There was no doubt about the fact that under the College Bill as well as under the Bill of the Central Committee, the management of the business, registration and everything else, would be in the hands of the nurses, and as far as that point was concerned there was no more merit in one Bill than in the other. There were only little differences between the two Bills, and he submitted it was time they ceased squabbling and tried to get into agreement.

The Chairman remarked that they were very much obliged to Professor Glaister for coming from Scotland to give them the benefit of his views, although they were in opposition to his (the Chairman's).

### Punishment for Offences.

Replying to a question, Professor Glaister said that in discussing the Bills it was very important that they should get their facts nailed to the counter. When a medical man committeed an offence his name was removed from the register. He had a right of appeal when questions of law were involved. He supposed nurses were capable of committing offences? If so, and there was a body in charge of the nursing profession, surely it was only right that the executive body should take cognisance of that offence, and if the nurse had offended against the law in such a way as made her not fit to remain a nurse, then that executive body ought to be charged with the removal of her name from the register. It might happen that a nurse so dealt with might think she was not getting justice, and, therefore, under the College Bill she was given the right of appeal to the High Court of Justice. He confessed that he did not see what more they could do. Clause 5 of the Bill was as clear as daylight on that point. The General Nursing Council was to have power under sub-section (g), "providing for the temporary or permanent removal from the register by the General Nursing Council of any nurse for such causes or offences, and after such inquiry as may be prescribed, but subject to the appeal provided for by this Act."

The Three Years' Certificate.

Mrs. Robertson Lawson said the question of a supplementary register in the College of Nursing Bill was only the difference between hard red-tape and an opening for progress in the future. But the question of a three years' certificate would never be changed. That with the College would be a foundation of membership which was as unalterable as the law of the Medes and Persians. The College might increase the years of regular training, but it would never shorten them.

An Appeal for Agreement.

Mrs. Gibson said she supposed she hardly had any right to speak because she was only just beginning to learn a little about the College of Nursing and the Royal British Nurses' Association. She thought, however, she might claim the privilege of saying just one word, as she was a trained nurse, trained under one of the best matrons in England—Miss Cox-Davies. She only wished to say that, after listening carefully to all the speeches, the differences between them seemed to be of such a minor character that she thought it was possible for both associations to meet together at a common table and agree on a Bill. (Cries of "No.")

Miss Breay said that going about the country she constantly came across matrons and nurses who had joined the College of Nursing without having even read its Articles of Association. Only the other day a matron told her she was a supporter of the College, and when she asked her whether she had read the Memorandum and Articles of Association she replied, "No, I have not seen them, but there are matrons on the College Council who I am sure won't approve of anything that is not right." On that ground she (Miss Breay) had no doubt that the nurses in that training-school would be asked to join the College, and in another case she knew of six nurses who went to take their initial training in a hospital where there was a College matron. They all told her they had joined the College—they did not know why, only matron had told them to "hurry up."

Important Matters of Principle.

Lieut.-Col. Goodall, replying on the discussion, remarked that Professor Glaister and Miss Breay said that those who were supporting the Central Committee were fighting about minor details. The whole point, however, was that they were not minor details for which they were fighting—they were important matters of principle that were going to affect the whole nursing profession in the future. If they had been minor details they would have agreed with the College long ago, but they were fundamental differences they were contending for. They were told: "Oh, anyhow the great thing is to get State registration, and you get that under the Council Bill." Their point, however, was that not only did they want State registration, but they wanted a proper State registration, and in their view they did not get that under the College Bill. He had said that the Bill of the Central Committee had been altered since it was first introduced eight or nine years ago. They were not above learning something from the College, just as they hoped the College was not above learning something from them, and they had no hesitation in saying that in such details as the registration of deaths of members of the nursing profession, which was an important matter, they had taken from the College Bill. They were told that in regard to supplementary registers the Bill of the College only proposed to take power to the General Nursing Council to institute them. They objected to that, for whatever the College or the new Council might do now, such a power opened the door in the future to the possibility of letting in all sorts of people. The College Bill opened the door too wide; but he supposed that was what Professor Glasier would only call a minor difference—they, however, considered that a very important detail. They had not heard from any of the speakers why it was necessary to have the College in the Bill at all; if the College really wanted State registration, why not let them have it without mentioning the College?

Mrs. Lawson: Because the College stands first and foremost for education and for registration.

Education and Registration.

Mrs. Bedford Fenwick said that she and her supporters stood for the organisation of the nursing profession by the State, and the organisation of the education of nursing by the State. That was what their Bill was. She was sorry that Professor Glaister was giving his valuable services to the draft of the opposition Bill, because she had very lively recollections of the delightful meetings they used to have when they were united when drafting the Central Committee's Bill. The whole thing she was concerned about was the protection of the trained nurse. The College Bill had no protection whatever in it for the trained nurse; but clause 19, which was one of the most important clauses in the Bill, and which she was glad to say was incorporated at her suggestion, did give a nurse the right of appeal to the High Court of Justice if she felt she had been unfairly dealt with. One of the very first anomalies in the College Bill was the incorporation of the College and its Memorandum of Association, which was in direct opposition to Clause 19.

Prof. Glaister: We have offered to withdraw clause 5 [3].

Mrs. Bedford Fenwick: But that would not alter the fact that if you incorporate the College under an Act of Parliament you have got to take the whole of its constitution with it. I am bound to say I have had many conversations in the past with Sir A. Stanley, and the wisest thing he ever said to me was, "I am not quite sure whether it is wise to incorporate the College in the Bill." That was in the early days—in 1915, when the first Bills were being drafted. We are quite sure now that it is not wise—(cheers)—and we hope that Sir A. Stanley will revert to his first instinct that it was unstatesmanlike, and that he will eliminate the College from the Bill.

Miss Cox-Davies remarked that Mrs. Lawson had said that the College stood for education first and registration second. As a very early worker for the College, and one still deeply interested in it, she would like to say that she took a happier view of its ideals. She thought that perhaps the best way to put it was to say that the College put registration and education hand in hand. Without registration they could not get education, and in her view education was quite as necessary for the nursing profession as registration.

Permissive Legislation—"A Blank Cheque."

The Chairman said he was sure they were much indebted to the various speakers who had come there that evening to explain the two Bills, and he hoped the conference would not be without its result. One strong objection which they took to the College Bill was that there was too much permissive legislation in it. He was a Scotchman, and he had a strong objection to giving blank cheques. The College Bill, as it stood now, was rather like giving a blank cheque both to the provisional and the permanent Council, and he had a very strong objection to giving blank cheques to anybody. If the Bill passed in its present form nurses would be in the position of not knowing what the Council was going to do in regard to their future regulations. They wanted those regulations laid down definitely as they were in their Bill—they wanted their powers and regulations to be laid down in such a manner that it should be plain what the Council could do and what it could not do. In conclusion, he was sure he was voicing all their feelings when he said that they heartily thanked all the speakers, and Professor Glaister especially, for coming all the way from Scotland to give them the benefit of their views. (Hear, hear.)

The meeting terminated with a vote of thanks to the Chairman for presiding.

